# Humoral response against COVID-19 in the population of western region of Poland

**DOI:** 10.3389/fpubh.2025.1648937

**Published:** 2025-08-05

**Authors:** Dominika Siedlecka, Aleksandra Ludziejewska, Lena Bielawska, Aleksandra Baszczuk, Marta Gawron, Mikołaj Danielewicz, Ewa Wysocka

**Affiliations:** ^1^Department of Laboratory Diagnostics, Poznan University of Medical Sciences, Poznan, Poland; ^2^Laboratory, Miedzyrzecz Hospital, Miedzyrzecz, Poland; ^3^Laboratory No. 2, Poznan University Clinical Hospital, Poznan, Poland

**Keywords:** anti-SARS-CoV-2 IgG antibodies, COVID-19, immunity, SARS-CoV-2, vaccine

## Abstract

**Background:**

Exposure to the SARS-CoV-2 activates two forms of active immunity: natural appearance after infection and vaccine induced immunity.

**Methods:**

We have conducted a retrospective analysis the serum concentration of IgG antibodies against SARS-CoV-2 in the population of Poznan University Clinical Hospital (PH) and Miedzyrzecz Hospital (MH) from 2021 to 2023.

**Results:**

In the entire study population (*n* = 3,104), as well as in the PH (*n* = 1,746) and MH (*n* = 1,358) groups, no significant differences in age (*p* = 0.1455) or BAU/ml values (*p* = 0.7874) between women and men were found. Significant differences were observed between the 18–35 and 36–60 age subgroups compared to the >60 age subgroup in the entire study population (*p* = 0.0022; *p* = 0.0001) and the PH group (*p* = 0.0176; *p* = 0.0003). In the MH group, significant differences were seen between the 18–35 and 36–60 age subgroups (*p* = 0.0305), as well as between the 18–35 and >60 age subgroups (*p* = 0.0050). A positive correlation was found between the number of infections and the number of tests conducted in each study group (R = 0.59, *p* = 0.0016).

**Conclusion:**

The concentration of anti-SARS-CoV-2 IgG antibodies is significantly higher in individuals over 60 years old compared to those in the 18–35 and 36–60 age groups. The correlations between age and antibody levels were significant but weak, suggesting that age should not be considered the main factor in predicting the immune response after vaccination or COVID-19 infection. Both women and men presented a similar immune responses to SARS-CoV-2 during the pandemic. Additionally, the number of infections within a specific time period influenced the number of individuals tested for anti-SARS-CoV-2 antibodies.

## 1 Introduction

COVID-19 is a disease whose etiological factor is the SARS-CoV-2 virus. The first case of infection was recorded in China in December 2019. Due to the occurrence of further epidemic outbreaks around the world, the World Health Organization (WHO) declared the COVID-19 pandemic on March 11, 2020. The increase in population immunity due to vaccinations and infections led to the announcement of the end of the pandemic on May 5, 2023 (1).

Since the start of the pandemic (as of May 17, 2024) 775,401,794 cases and 7,047,316 deaths due to COVID-19 were recorded worldwide. WHO has monitored changes in the spread of the COVID-19 disease in the six regions: the European Region, the Region of the Americas, the South-East Asia Region, the African Region, the Western Pacific Region and the Eastern Mediterranean Region. On a global scale, based on confirmed cases, the highest morbidity and mortality are observed on the European continent. However, the lowest number of infections and deaths were recorded in Africa (2).

**Figure 1:**
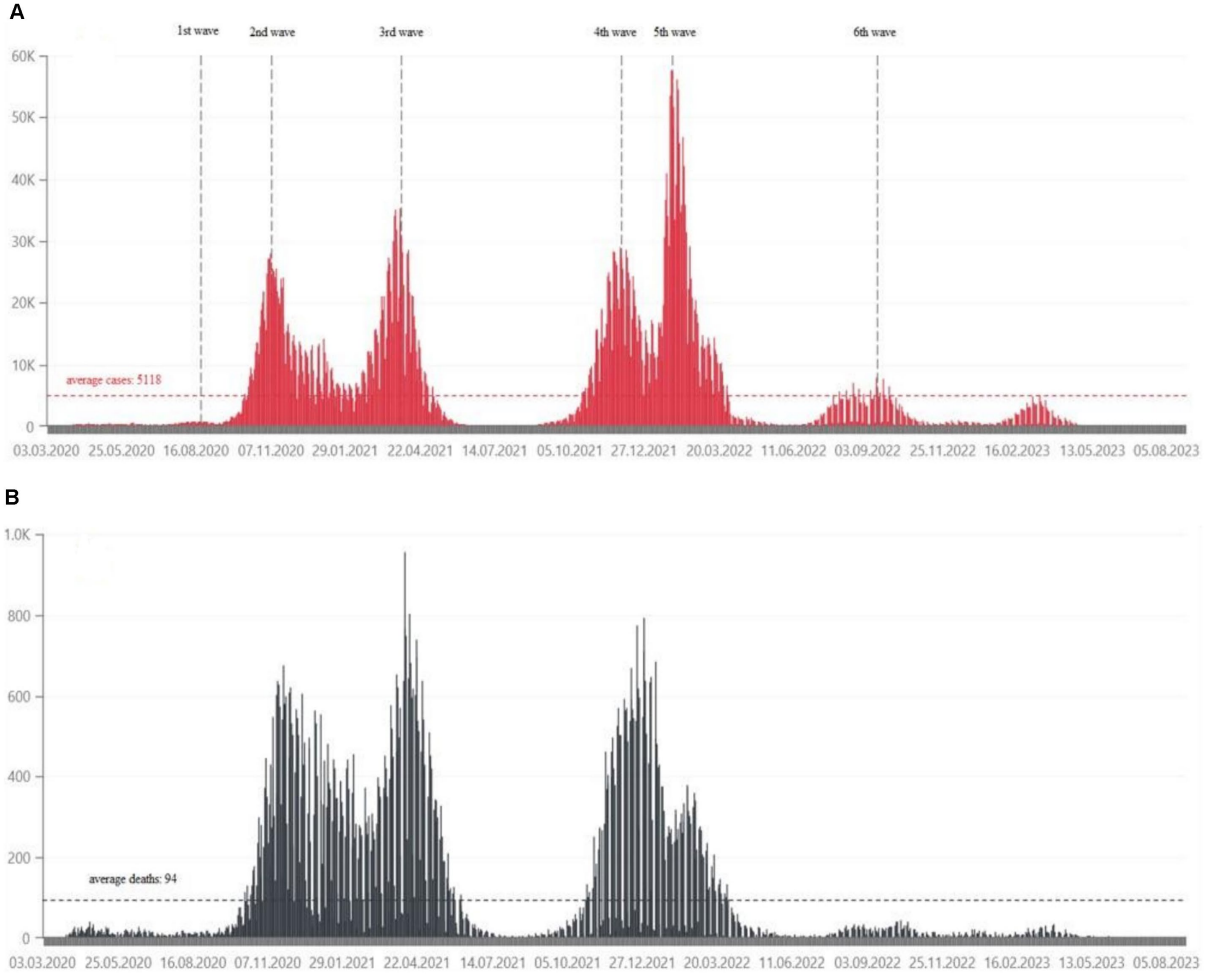
**(A)** Daily confirmed COVID-19 cases in Poland, from March 2020, in tens of thousands of people (21). **(B)** Daily deaths due to COVID-19 in Poland, from March 2020, in hundreds of people (21).

**Figure 2:**
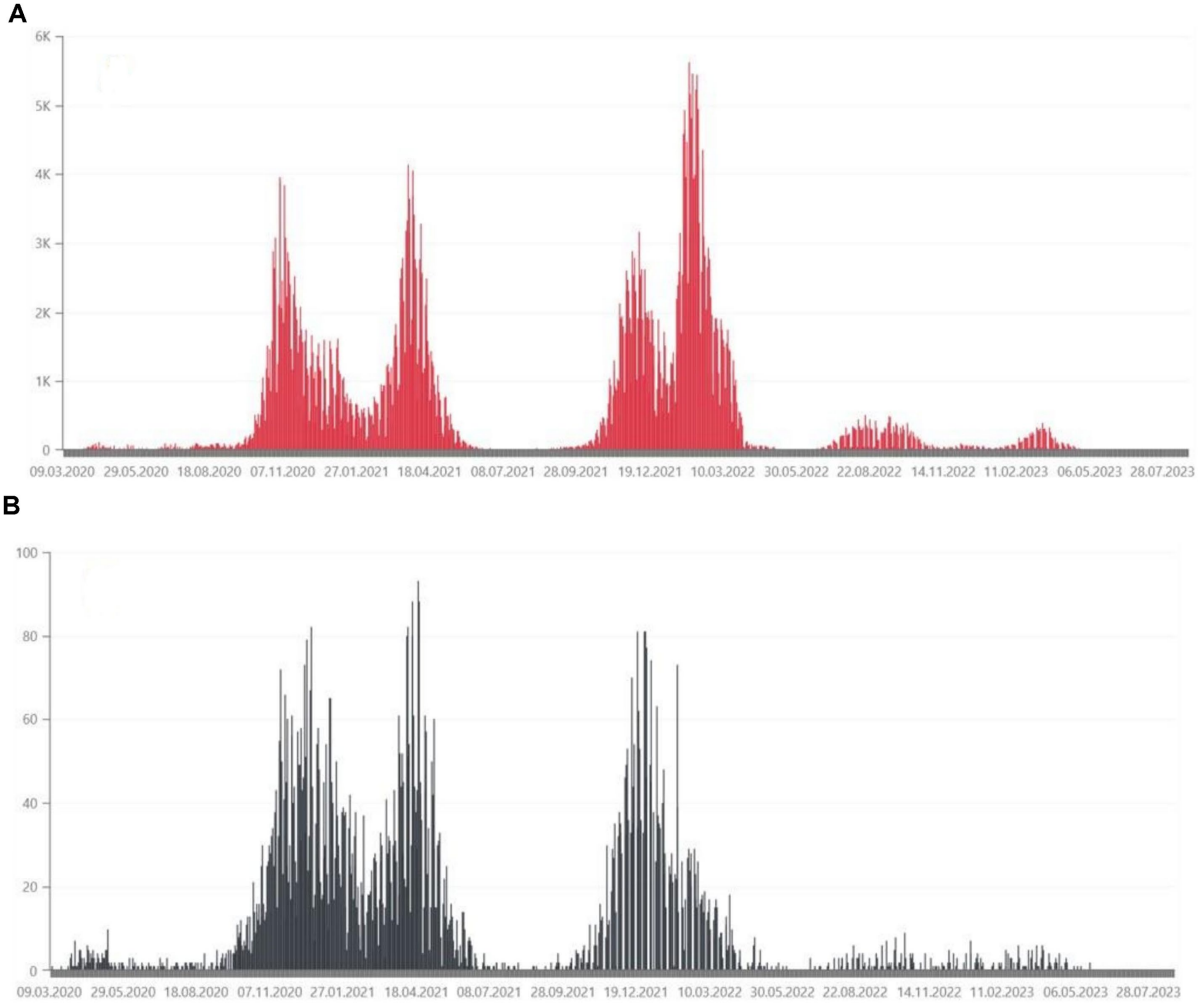
**(A)** Daily confirmed COVID-19 cases in the Wielkopolskie Voivodship, from March 2020, in thousands of people (35). **(B)** Daily deaths due to COVID-19 in the Wielkopolskie Voivodship, from March 2020, in tens of people (35).

**Figure 3:**
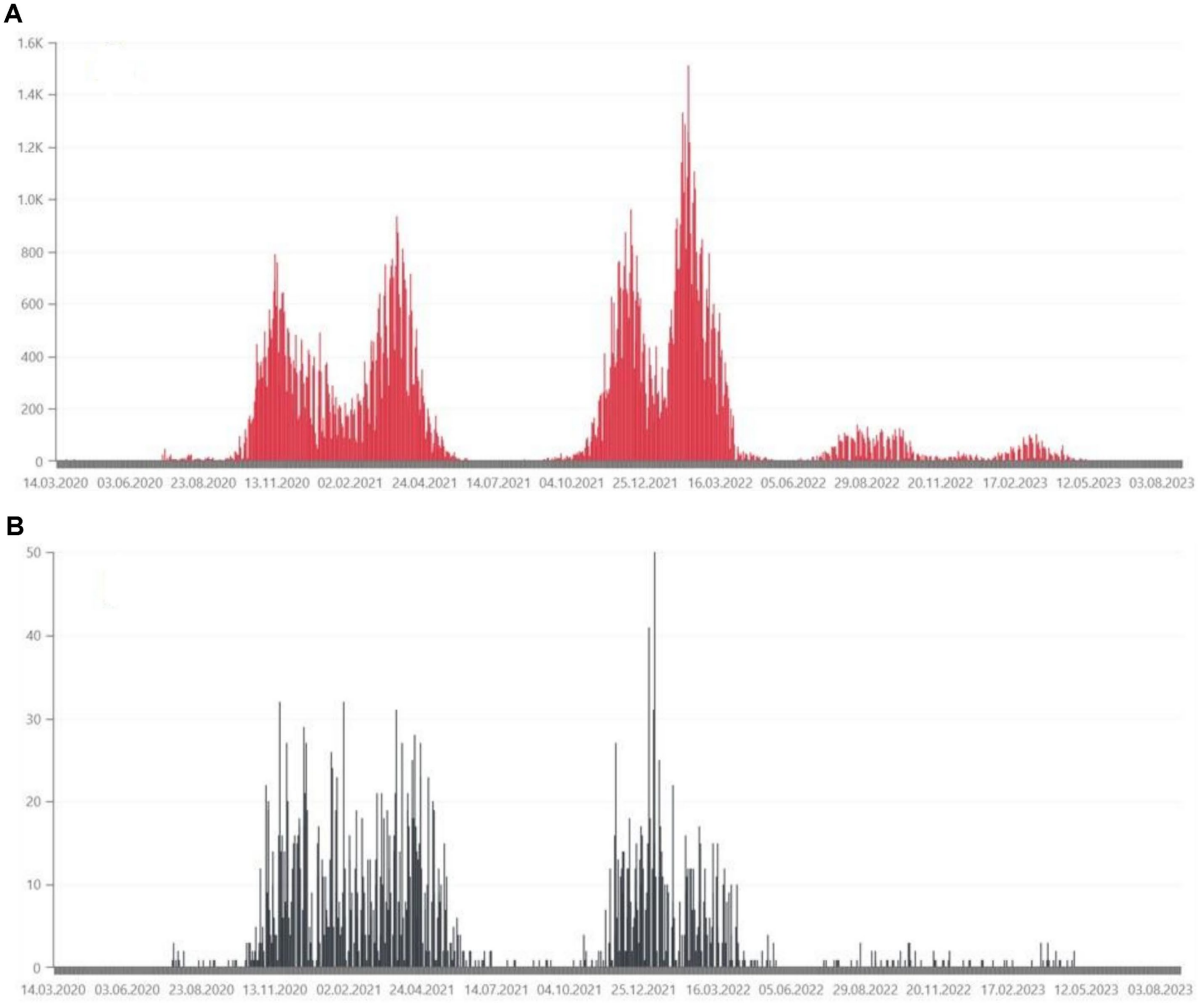
**(A)** Daily confirmed COVID-19 cases in the Lubuskie Voivodeship, from March 2020, in hundreds of people (36). **(B)** Daily deaths due to COVID-19 in the Lubuskie Voivodeship, from March 2020, in tens of people (36).

From March 4, 2020 to May 28, 2024, the Ministry of Health has registered 6,662,958 SARS-CoV-2 infections in Poland, including re-infections in 3.48% of cases. At that time, 120,602 deaths were recorded (3). The daily number of confirmed cases and deaths due to COVID-19 in Poland are shown in Figure 1. Statistical data on confirmed infections and deaths in the Wielkopolskie (Figure 2) and Lubuskie Voivodships (Figure 3) are also presented. Wielkopolska is the third most populous voivodship in Poland, whereas the Lubuskie is one of the most sparsely populated.

Exposure to the SARS-CoV-2 virus in the upper respiratory tract initiates the activation of the immune pathway. The virus enters host cells by binding spike (S) glycoproteins on the outer surface of SARS-CoV-2 to ACE2 (angiotensin converting enzyme 2), which acts as a receptor for SARS-CoV-2. COVID-19 contributes to innate and acquired immune responses: CD8+ T cells directly kill virus-infected cells (cellular immune response) and CD4+ T cells stimulate B cells to produce anti-SARS-CoV-2 antibodies (humoral immune response) (4–6). The immunodominant epitopes, against which B and T cell respond, carries the S-protein of SARS-CoV-2 (7). Early after infection are detected S-specific IgM, IgA and IgG antibodies. IgG antibody levels and IgG memory B cells can persist a longer time post-infection or vaccination (8, 9).

There are two forms of active immunity: natural appearance after infection and vaccine-induced immunity (10, 11). Currently, the European Commission, extended authorization for two mRNA and two protein vaccines (12–19).

The aim of this study was to analyze the serum concentration of IgG antibodies against SARS-CoV-2 in the population of Wielkopolskie and Lubuskie Voivodships. In addition, it was intended to investigate the impact of age and gender on the humoral immune response and the correlations between examined parameters.

## 2 Materials and methods

### 2.1 Study population

The study was conducted based on a retrospective analysis of medical records of the Poznan University Clinical Hospital, previously Poznan University Hospital of Lord’s Transfiguration (Wielkopolskie Voivodeship) - PH and Miedzyrzecz Hospital (Lubuskie Voivodeship) - MH from 2021–2023. There was the general population of people who were hospitalized for reasons other than COVID-19, who came from a clinic or on their own. COVID-19 disease or vaccination were not taken into account. The study group consisted of 3,104 people (1,747 female and 1,357 male), including newborns and people aged 97. The size of the study group divided into voivodeships and age, and gender subgroups is presented in the Table 1. Due to significant disproportions between number of children and adults, in studied groups, it was decided that the analysis of the results, apart from the correlation between the number of documented infections and the number of tests, would be conducted only in the group of adults. Taking into account the existence of tests from different manufacturers examining the concentration of anti-SARS-CoV-2 IgG antibodies, the results of our tests were expressed in the international unit - BAU/ml to enable their comparison between study groups.

**Table 1:** The size of entire study population, PH and MH groups, including age and gender.

Study groups	The entire study population	PH group	MH group
All	3,104	1,746	1,358
Women	1,747	1,006	741
Men	1,357	740	617
Children	936	30	906
**Adults**	**2,168**	**1,716**	**452**
**Adult women**	**1,287**	**995**	**292**
**Adult men**	**881**	**721**	**160**

The bold values are the size of study group taken into account in further statistical analysis. Is the size of study group without children.

### 2.2 Measurements and analysis

It was documented in the healthcare centers mentioned above, that blood was collected in accordance with current standards, both in terms of patient and sampling. Whole blood was collected into a clotting activator tube (4.9 mL) for serum preparation (S Monovette®, Sarstedt, Germany).

The concentration of IgG antibodies against the receptor-binding domain (RBD), S1 subunit of the SARS-CoV-2 virus spike protein in the serum of patients was measured using the indirect chemiluminescence method used the “sandwich” principle.

In the PH (Wielkopolskie Voivodeship) the concentration of anti-SARS-CoV-2 IgG antibodies was measured on the Atellica® IM Analyzer (Siemens Healthcare Diagnostics Inc., USA).

The sensitivity of the test was determined for material collected after a certain number of days after obtaining a positive PCR test result and was: 0–6 days after PCR - 50.82%; 7–13 days after PCR - 82.47%; 14–20 days after PCR - 91.14%; ≥ 20 days after PCR - 96.41%.

Specificity was determined based on samples collected before the outbreak of the COVID-19 pandemic and was estimated at 99.90%.

The test results are given in the form of an index value with the designation of non-reactive or reactive: non-reactive: index value < 1.00 (no antibodies present); reactive: index value ≥ 1.00 (presence of antibodies).

The measurement range was 0.50–150.00 index values, with an index value of 1.00 corresponding to 1.00 U/mL. Using the conversion BAU/ml = U/ml x 21.8, antibody test results from the PH group are given in the BAU/ml unit.

In the MH (Lubuskie Voivodeship) the concentration of anti-SARS-CoV-2 IgG antibodies was measured on the COBAS® analyzer (Roche Diagnostics, UK).

The sensitivity of the test was determined on the basis of samples of patients showing symptoms of COVID-19 disease, confirmed by PCR testing, and was 98.8%.

Specificity based on patient samples obtained before October 2019 was 99.91%.

The test results are given with the designation of negative or positive: negative:< 0.80 (no antibodies present); positive: ≥ 0.80 (presence of antibodies).

The measurement range was 0.40–250 U/mL. Using the conversion BAU/ml = U/ml x 0.972, the results of antibody tests from the MH group were given in the BAU/ml unit.

### 2.3 Statistical methods

The statistical analysis was performed using the Statistica 13.3 software (StatSoft Inc., Tulsa, OK, USA) and PQStat Software v.1.8.4 (Poznan, Poland). The Shapiro–Wilk test was used to verify the normality of data distribution. In the absence of a normal distribution, non-parametric tests were used in further analysis. All results were expressed as median and interquartile range. The Mann–Whitney U test was used to assess the significance of differences between the two groups, while the comparisons of many groups were performed using the Kruskal-Wallis test with *post hoc* analysis of multiple comparisons. Correlations between the studied variables were assessed using Spearman’s R coefficient.

## 3 Results

### 3.1 Descriptive characteristics of the study population

**Figure 4:**
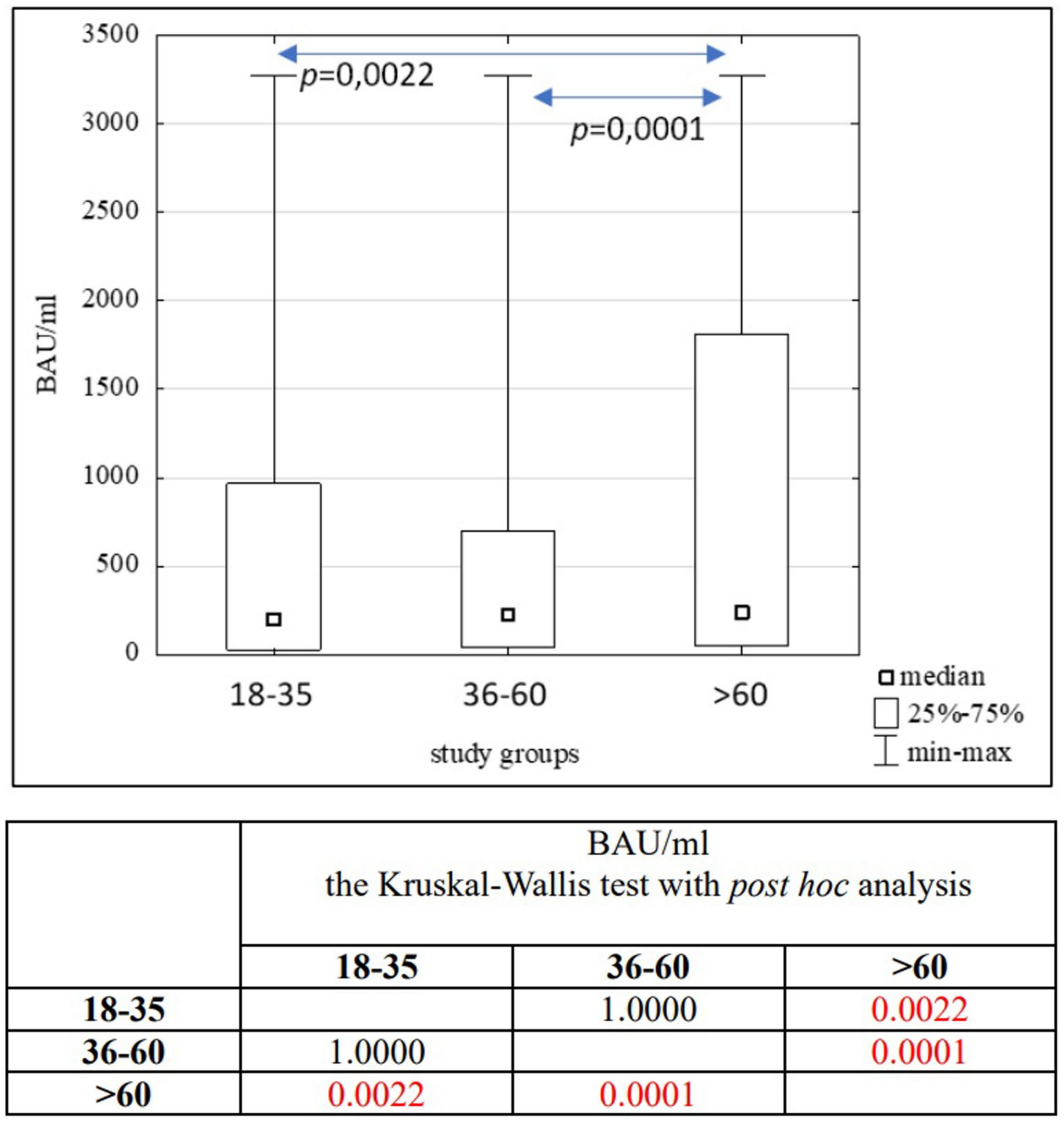
The comparison of BAU/ml between age subgroups in the entire study population.

**Figure 5:**
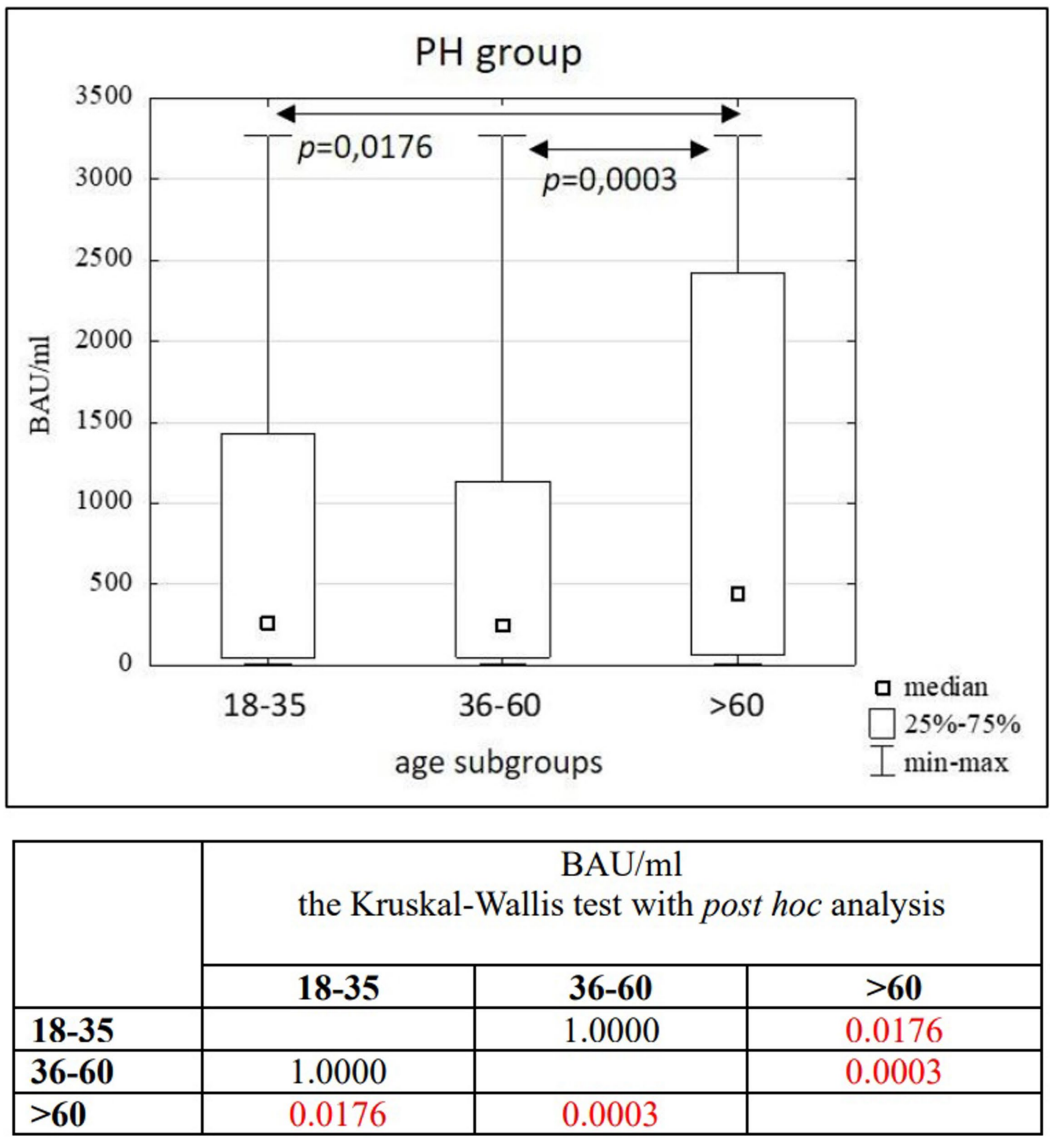
The comparison of BAU/ml between age subgroups in the PH group.

**Figure 6:**
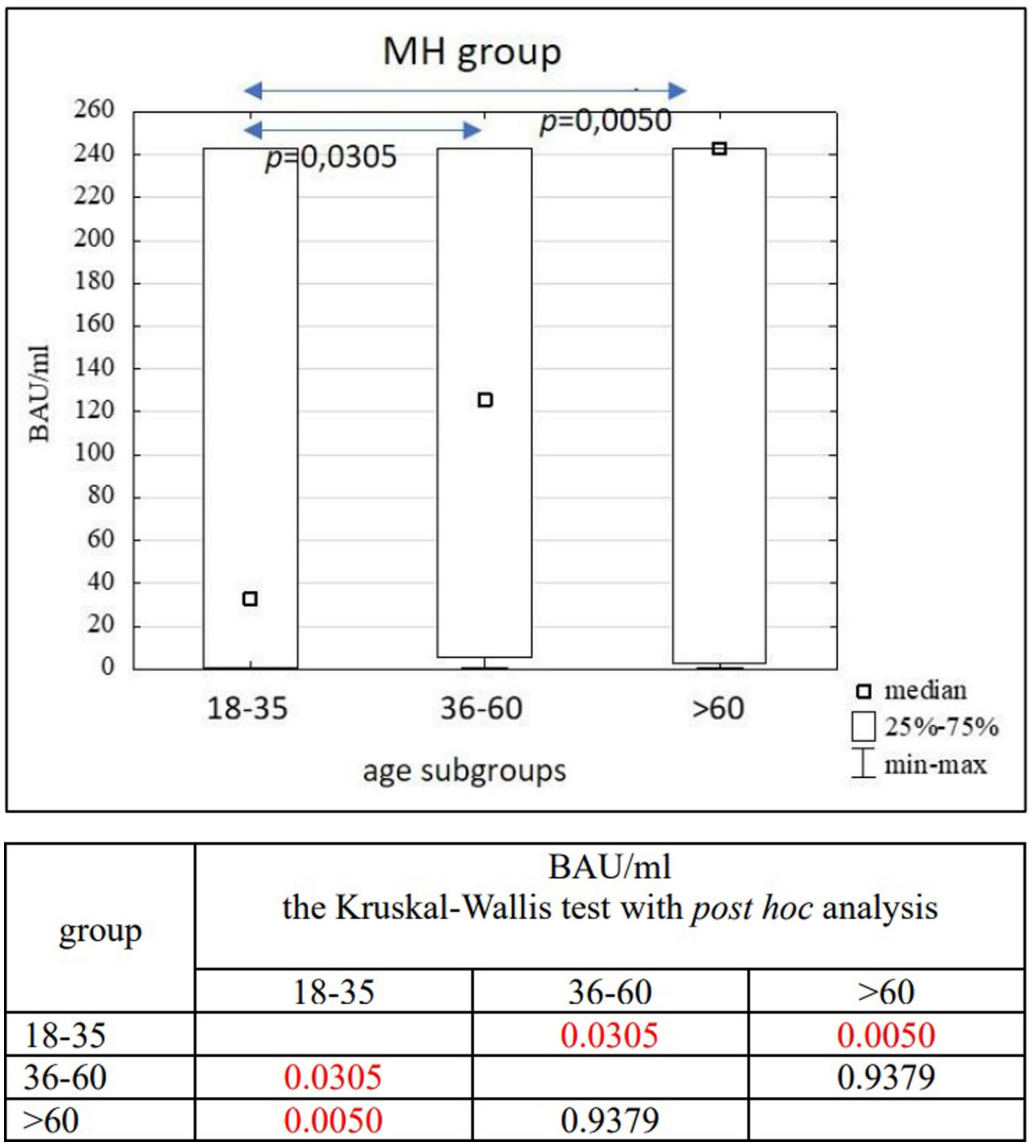
The comparison of BAU/ml between age subgroups in the MH group.

The statistical analysis of BAU/ml values and age was performed in the entire study population (*n* = 2,168) and separately in the PH group (*n* = 1,716) and the MH group (*n* = 452) as well. Each group was also divided into subgroups of women and men, and into three age subgroups: 18–35, 36–60, and >60 years old. The analysis results in the entire study population are presented in Tables 2, 3 and Figure 4. The results in the PH and MH groups show Tables 4–7 and Figures 5, 6.

**Table 2:** The characteristics of age and BAU/ml values in the entire study population and the comparison of the subgroups of women and men.

Parameter	The study population *n* = 2,168	Women *n* = 1,287	Men *n* = 881	*p*
Age	52.00 38.00–64.00	52.00 38.00–63.00	53.50 38.00–65.00	0.1455 (ns)
BAU/ml	243.00 39.47–994.40	243.00 42.10–917.10	243.00 34.00–1110.50	0.7874 (ns)

Data are presented as median and interquartile range.

n, group multiplicity; ns, nonsignificant.

**Table 3:** The characteristics of age and BAU/ml values in the age subgroups: 18–35, 36–60 and >60 years old in the entire study population.

Parameter	18–35 *n* = 454	36–60 *n* = 1,018	>60 *n* = 696	*p*
Age	28.50 24.50–32.00	49.00 42.00–55.50	68.00 64.50–73.50	by definition
BAU/ml	204.15 28.30–967.92	229.68 39.24–701.30	243.00 51.12–1806.94	<0.00001

Data are presented as median and interquartile range.

n, group multiplicity.

**Table 4:** The characteristics of age and BAU/ml values in the PH group and the comparison of the subgroups of women and men.

Parameter	The study population *n* = 1,716	Women *n* = 995	Men *n* = 721	*p*
Age	52.50 38.00–64.50	52.00 38.00–63.00	55.00 37.00–66.00	0.1037 (ns)
BAU/ml	213.27 52.76–1606.75	309.80 60.40–1575.50	334.80 44.50–1665.52	0.6740 (ns)

Data are presented as median and interquartile range.

n, group multiplicity; ns, nonsignificant.

**Table 5:** The characteristics of age and BAU/ml values in the age subgroups: 18–35, 36–60 and >60 years old in the PH group.

Parameter	18–35 *n* = 368	36–60 *n* = 786	>60 *n* = 562	*p*
Age	28.50 25.00–32.00	49.50 43.00–56.00	68.50 65.00–74.00	by definition
BAU/ml	263.58 48.05–1431.95	258.55 49.90–1132.50	443.00 67.58–2418.30	0.0003

Data are presented as median and interquartile range.

n, group multiplicity.

**Table 6:** The characteristics of age and BAU/ml values in the MH group and the comparison of the subgroups of women and men.

Parameter	The study population *n* = 452	Women *n* = 292	Men *n* = 160	*p*
Age	51.00 39.00–62.00	51.00 38.00–62.00	50.50 39.00–63.00	0.8722 (ns)
BAU/ml	125.19 0.39–243.00	133.07 1.24–243.00	123.40 0.39–243.00	0.5138 (ns)

Data are presented as median and interquartile range.

n, group multiplicity; ns, nonsignificant.

**Table 7:** The characteristics of age and BAU/ml values in the age subgroups: 18–35, 36–60 and >60 years old in the MH group.

Parameter	18–35 *n* = 86	36–60 *n* = 232	>60 *n* = 134	*p*
Age	30.00 22.00–33.00	47.00 41.00–55.00	66.50 63.00–71.00	by definition
BAU/ml	33.01 0.39–243.00	125.73 5.52–243.00	243.00 2.91–243.00	0.0008

Data are presented as median and interquartile range.

n, group multiplicity.

In the entire study population and the PH and MH groups, no difference in age or BAU/ml values between women and men was observed. The comparison of the analyzed age subgroups (18–35, 36–60, >60) in terms of BAU/ml, revealed differences between them in each study groups. In the entire study population and the PH group, the subgroups 18–35 and 36–60 years old differed significantly from the subgroup >60 years old, in which BAU/ml reached the highest values. In the MH group, a difference between the subgroups 18–35 and 36–60 years old, as well as 18–35 and >60 years old, was observed. The values of BAU/ml increased in subsequent subgroups.

### 3.2 The characteristics of the entire study population

The characteristics of the entire study population are shown in Tables 2, 3 and Figure 4.

#### 3.2.1 The characteristics of the PH study group

The characteristics of the PH study group are shown in Tables 4, 5 and Figure 5.

#### 3.2.2 The characteristics of the MH study group

The characteristics of the MH study group are shown in Tables 6, 7 and Figure 6.

#### 3.2.3 The analysis of the number of positive and negative test results in the study populations

Tables 8–10 show the percentage of positive and negative results of the SARS-CoV-2 antibody test obtained in the entire study population and in all subgroups. In most study groups, positive results were approximately 80%. The lowest percentage was 65.12% among subjects aged 18–35 in the MH group, and the highest was 83.92% among women in the PH group.

**Table 8:** The percentage of positive and negative results of the SARS-CoV-2 antibody test in entire study population and subgroups.

Study groups	Negative test result	%	Positive test result	%
The study population (*n* = 2,168)	416	19.19	1,752	80.81
Women (*n* = 1,287)	232	18.03	1,055	81.97
Men (*n* = 881)	184	20.88	697	79.12
18–35 (*n* = 454)	95	20.93	359	79.07
36–60 (*n* = 1,018)	196	19.25	822	80.75
>60 (*n* = 696)	125	17.96	571	82.04

**Table 9:** The percentage of positive and negative results of the SARS-CoV-2 antibody test in the PH group and subgroups.

Study groups	Negative test result	%	Positive test result	%
The study population (*n* = 1,716)	302	17.60	1,414	82.40
Women (*n* = 995)	160	16.08	835	83.92
Men (*n* = 721)	142	19.69	579	80.31
18–35 (*n* = 368)	65	17.66	303	82.34
36–60 (*n* = 786)	144	18.32	642	81.68
>60 (*n* = 562)	93	16.55	469	83.45

**Table 10:** The percentage of positive and negative results of the SARS-CoV-2 antibody test in the MH group and subgroups.

Study groups	Negative test result	%	Positive test result	%
The study population (*n* = 452)	114	25.22	338	74.78
Women (*n* = 292)	72	24.66	220	75.34
Men (*n* = 160)	42	26.25	118	73.75
18–35 (*n* = 86)	30	34.88	56	65.12
36–60 (*n* = 232)	52	22.41	180	77.59
>60 (*n* = 134)	32	23.88	102	76.12

#### 3.2.4 The analysis of the number of SARS-CoV-2 virus infections and the number of SARS-CoV-2 IgG antibody tests performed

**Figure 7:**
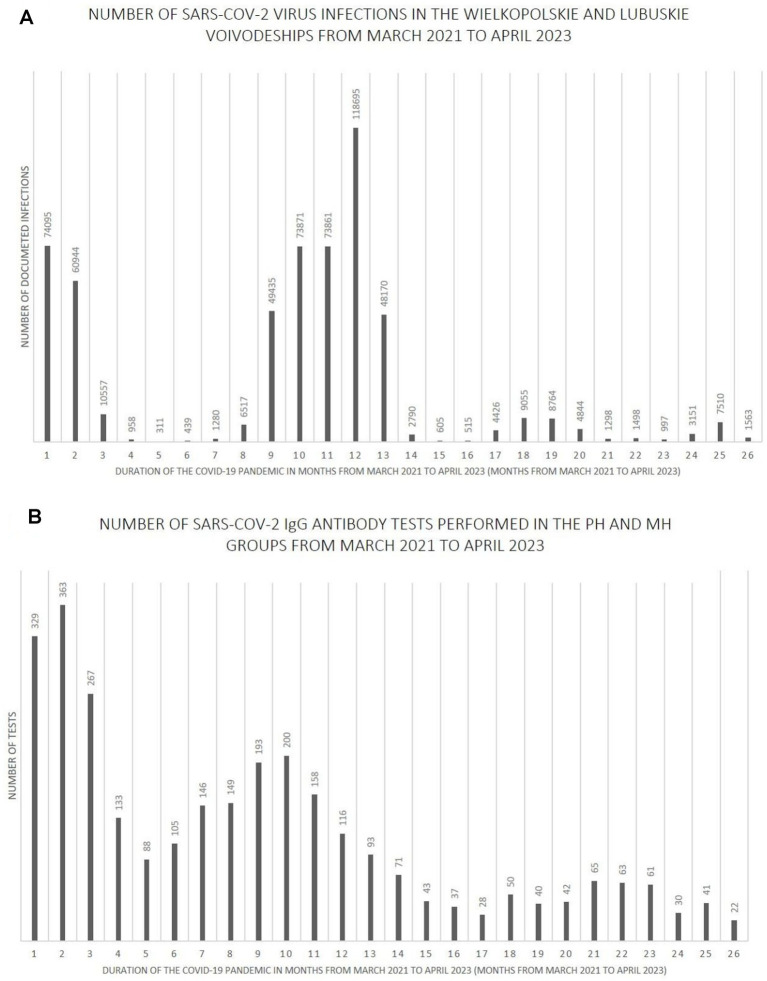
**(A)** The number SARS-CoV-2 virus infections in the Wielkopolskie and Lubuskie Voivodeships from March 2021 to April 2023. **(B)** The number SARS-CoV-2 IgG antibody tests performed in the PH and MH groups from March 2021 to April 2023.

**Figure 8:**
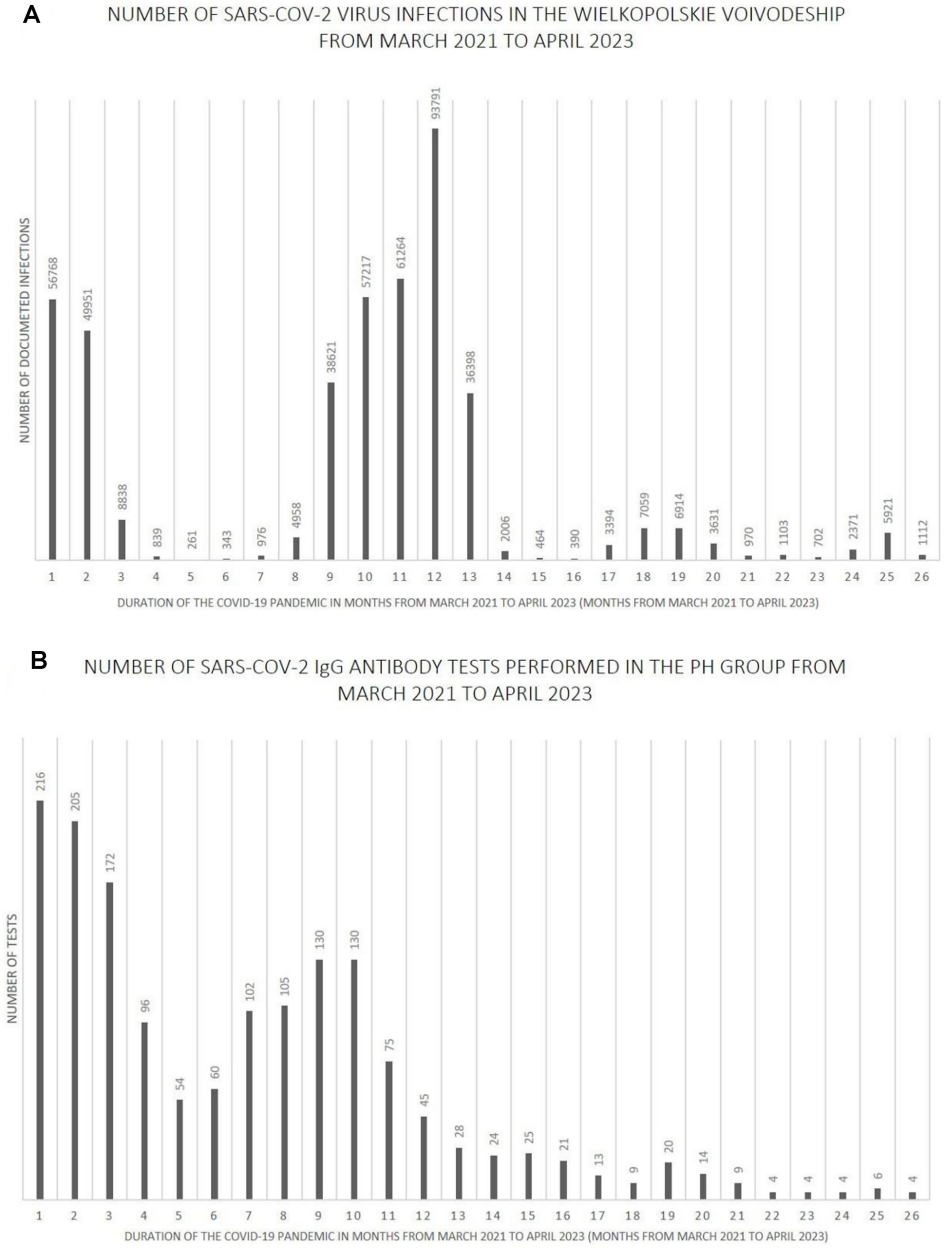
**(A)** The number SARS-CoV-2 virus infections in the Wielkopolskie Voivodeship from March 2021 to April 2023. **(B)** The number SARS-CoV-2 IgG antibody tests performed in the PH group from March 2021 to April 2023.

**Figure 9:**
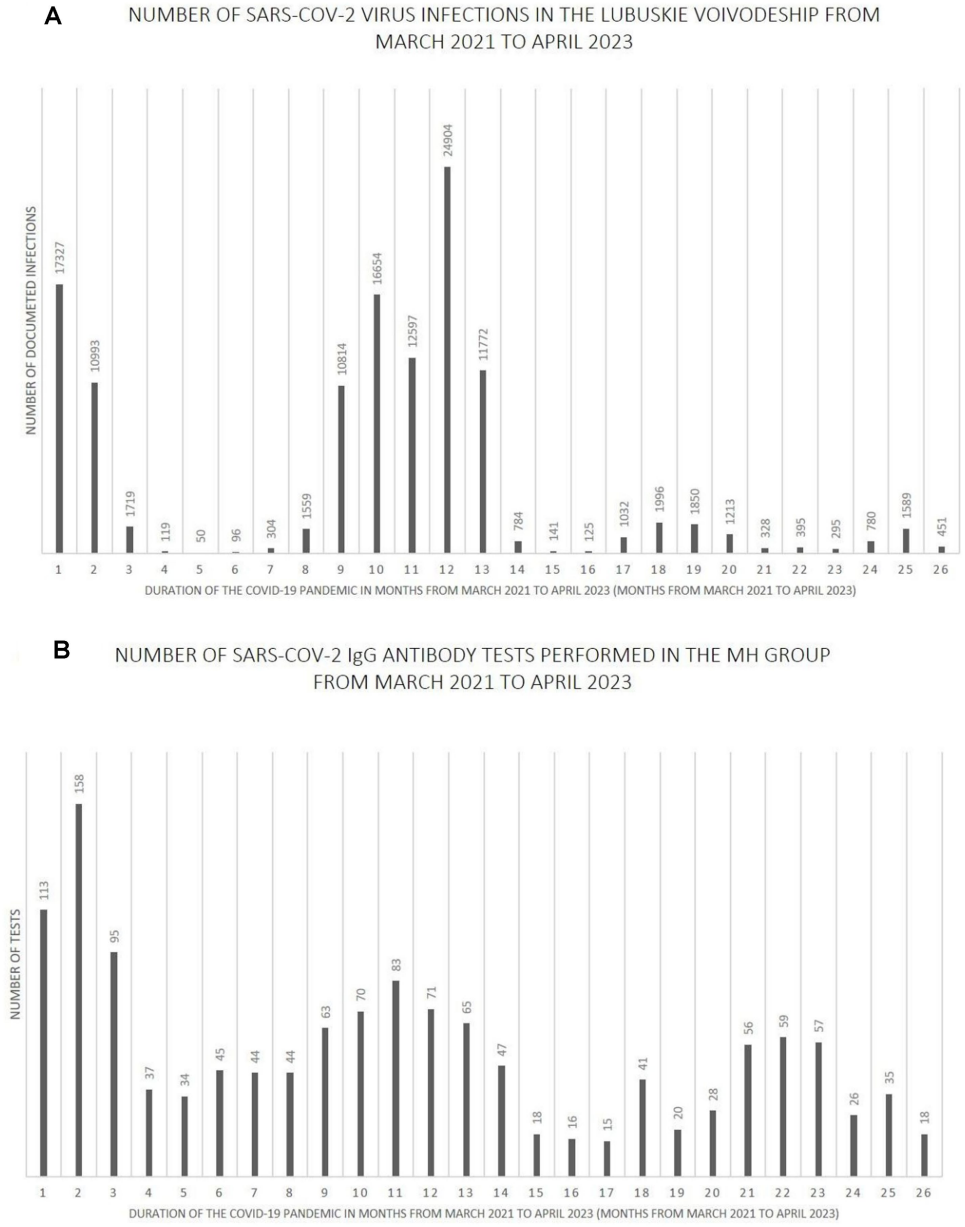
**(A)** The number SARS-CoV-2 virus infections in the Lubuskie Voivodeship from March 2021 to April 2023. **(B)** The number SARS-CoV-2 IgG antibody tests performed in the MH group from March 2021 to April 2023.

Figures 7–9 show the number of documented SARS-CoV-2 virus infections in the following months from March 2021 to April 2023 in the Wielkopolskie and Lubuskie Voivodeships in total (Figure 7A) and separately (Figures 8A, 9A). Figures 7B, 8B, 9B show the number of SARS-CoV-2 IgG antibody tests performed in the study groups, respectively.

Further analysis showed statistically significant positive correlations between the number of infections and the number of tests performed in each study group. The strength of the correlation was the highest in the MH group (Spearman’s *R* = 0.59 and *p*-value = 0.0016). Spearman’s R coefficient and p-value in the entire study population and the PH group were *R* = 0.46, *p* = 0.0172, and *R* = 0.40, *p* = 0.0419, respectively.

#### 3.2.5 The correlation analysis between BAU/ml values and age

The results of the correlation analysis between age and BAU/ml values in the study groups and subgroups are presented in Tables 11–13. In the entire study population, statistically significant positive correlations were observed in each subgroup, but their strength was weak. In the PH and MH groups, correlations between examined parameters were also found in most subgroups, but similarly to the entire population, their strength was also weak.

**Table 11:** Correlations between age and BAU/ml values in the entire study population and subgroups.

Study groups	Spearman’s R	*p*
The study population (*n* = 2,168)	0.11	<0.00001
Women (*n* = 1,287)	0.10	0.0003
Men (*n* = 881)	0.12	0.0005
18–35 (*n* = 454)	−0.11	0.0204
36–60 (*n* = 1,018)	0.11	0.0004
>60 (*n* = 696)	0.12	0.0006

**Table 12:** Correlations between age and BAU/ml values in the PH group and subgroups.

Study groups	Spearman’s R	*p*
The study population (*n* = 1,716)	0.10	0.0001
Women (*n* = 995)	0.08	0.0121
Men (*n* = 721)	0.11	0.0026
18–35 (*n* = 368)	−0.10	0.0676 (ns)
36–60 (*n* = 786)	0.06	0.0726 (ns)
>60 (*n* = 562)	0.12	0.0037

**Table 13:** Correlations between age and BAU/ml values in the MH group and subgroups.

Study groups	Spearman’s R	*p*
The study population (*n* = 452)	0.18	0.0001
Women (*n* = 292)	0.21	0.0004
Men (*n* = 160)	0.13	0.1128 (ns)
18–35 (*n* = 86)	−0.24	0.024
36–60 (*n* = 232)	0.24	0.0003
>60 (*n* = 134)	−0.03	0.6935 (ns)

## 4 Discussion

After the outbreak of the COVID-19 pandemic on a global scale, one of the elements of quick response to this phenomenon was the initiation of research, thus enabling deeper understanding of the pathogen, the disease it causes, as well as human body’s response to the new threat. The earliest research on the production of antibodies against SARS-CoV-2 appeared already in 2020 and the trend of conducting this type of tests continues to this day. This article aims to clarify emergence of immunity to this virus in a specific place and time, as well as the relationship between said immunity and various aspects of researched populations.

In our case, studied population included 2,168 adult participants in various age groups, whose tests were carried out in the Lubuskie and Wielkopolskie Voivodeships, in the period from March 2021 to April 2023. A more detailed examination of the characteristics of those participants allows us to conclude that there is relationship between the appearance of specific concentrations of anti-SARS-CoV-2 antibodies and periods of subsequent waves of infection. Those waves were also followed by mass testing of people. Over the following years, there is a clear positive trend in the concentration of immune antibodies against this virus. This is, of course, not a phenomenon isolated to Poland, as similar conclusions emerge from publicly available data from the Office of National Statistics, which regularly published the results of tests determining the concentration of antibodies in Great Britain, Wales, and Scotland (20). As these countries went through subsequent waves of the pandemic, the number of citizens in different age groups, showing higher concentrations of antibodies increased, compared to the previous months.

In addition to the natural immunity that the test subjects acquired throughout the study, we cannot forget about the cases of patients with immunity resulting from receiving at least 1 dose of the anti- COVID-19 vaccine, which in Poland became publicly available from the beginning of 2021, first for health care workers, older adult and later on- for entire population.

The results collected for this study extend from 2021 to 2023. When discussing them, one should take into account the initial predominance of natural immunity cases, which gradually gave way to the so-called hybrid immunity, occurring in cases, where people who have already went through COVID-19 received vaccinations as well. The number of vaccinated people increased in the years 2021–2023 to approximately 65% of the Polish population (21). Considering that only adults are taken into account in this work, vast majority of the study group most likely showed hybrid immunity, especially among people whose data we collected in 2022 and 2023. This explains the high number of people showing positive test result of SARS-CoV-2 antibody in our work- 80.81% in the entire study group; 82.4% in PH and 74.78% in MH.

Our results are very similar to those obtained by Jones et al. (22), studying the blood donor population in the United States. That team also recognized the positive effects of vaccines and the aforementioned hybrid immunity. They estimated, that in the second quarter of 2021, 68.4% of individuals in the surveyed US population aged ≥16 years had SARS-CoV-2 antibodies induced by infection or vaccination, including only 8.9% achieving hybrid immunity. However, the percentage of results indicating hybrid immunity had increased by the third quarter of 2022 and was already 47.7%, while entire number of positive results was 96.4%.

It would be appropriate to also mention a high percentage of immune patients in a study presented by the Norwegian Institute of Public Health (23). Based on samples collected from 1,914 people in 2022, ranging in age from infants to older adult, it was estimated that in August 2022 97% of the country’s population had antibodies to SARS-CoV-2.

Despite that, our results in immunity percentage are slightly lower than those from Norway or the US it is important to mention that our study group was a bit more tightly defined, as it came from two provinces - Lubuskie and Wielkopolskie.

In contrast to our findings, there are articles showcasing poorer results of seropositivity through certain populations, before wide spread of vaccines in Europe. We can mention the study by Rebhoz et al. (24) of antibody levels in the Wachau region of Lower Austria, where positive results for IgG antibodies against COVID-19 in June 2020 accounted for only 8.5% of individuals. In February 2021, this number rose to 25%.

The other study, conducted in France by Decarreaux’s team covered three waves of the pandemic, collecting samples from adult volunteers since November 2020 to July 2021 (25). Lower results were observed at the beginning months - 11.5% seropositivity with an average antibody concentration of 86.6 BAU/ml. It was only after May 2021, when amount of positive results increased to 68.1%.

To summarize this part of the topic - most studies of population immunity to SARS-CoV-2 have indicated a clear correlation between reactive results for specific antibodies and the passage of a wave of infection with this virus through a region, as well as a clear increase in immunity in the population due to the gradual introduction of subsequent vaccines. The average concentration of mentioned antibodies in our studied population equaled 243 BAU/ml and the highest concentration was 994.4 BAU/ml. It is unlikely such results would occur without both natural as well as post-vaccine immunity of our patients.

As previously presented, the median age of our study group was 52 years and the subgroups of subjects from different voivodeships were appropriately similar to use them to compare the influence of age on the antibody concentration results achieved by patients using Kruskal-Wallis test. It was noticeable that older people achieved, on average, higher results than other subgroups. This is an interesting event considering the gradual general weakening of the immune system that comes with age, which would suggest a completely opposite relationship. This could be due to the fact that it was people in older age groups, who belonged to the so-called “vulnerable groups,” who may have received vaccines earlier than, for example, young adults.

It should also be mentioned that in the MH group there was a statistically significant relationship between the 18–35 subgroup and the other two age subgroups, where the youngest people had the lowest antibody concentration results. Again it is a non-intuitive result, but also potentially explained by the fact that people in this age range were the last to receive subsequent doses of vaccines. In addition, such results could have been influenced by certain social and behavioral differences between age groups which “protected” younger people from repeated COVID-19 cases, which would have resulted in no increase in the number of antibodies.

Spearman’s correlation test between age and antibody against SARS-CoV-2 concentration in various subgroups used in the test confirmed a positive correlation in most cases, although it was statistically weak - a stronger correlation could be achieved using the results collected from larger number of volunteers (in our case *n* = 2,168).

Interestingly, Yang and his research team, came to similar conclusions on the topic of the connection between age and the reaction to SARS-CoV-2 infection (26). In their work, the level of anti-SARS-CoV-2 IgG showed a moderate, but positive correlation with age in adults (*R* = 0.24, *p* < 0.001). This study used a large amount of data in statistical analysis- 31,426 test results from both adult and pediatric patients from New York.

There are also works, which in contrast to our results, showcase a negative correlation between antibody levels and age. Such conclusions appear in articles by Markewitz et al. (27) and Papaneophytou et al. (28). Moreover, Weidner et al. (29) noted the appearance of more frequent immune response in the group of 18–25 year-olds than in other age groups.

Some teams, like Kasztelewicz et al. (30) and Rastawicki et al. (31) did not note any correlation between age and antibody levels in the individuals studied.

In conclusion, the results of our study, as well as other existing in recent literature suggest that there are different profiles of SARS-CoV-2 virus-specific antibody responses and they show age-dependent differences. But that idea still needs further research, as different sources showcase contradictory.

According to the statistical tests done with collected data, the gender of the participants in the study had no significant effect on the concentration of detected antibodies (*p* < 0.05). The same goes for the ratio of positive to negative results for men and women. These results were consistent for both study groups – PH and MH, as well as the entire study population. Thus, assumption that female and male participants responded similarly to the changing pandemic environment seems appropriate. Reflecting on our findings, vaccines and natural response to SARS-CoV-2 infection are resulting in similar boost of specific antibodies’ titers and COVID resistance, no matter the gender.

This conclusion has already appeared in the literature, including the work of the teams Markewitz et al. (27), Weidner et al. (29), Kasztelewicz et al. (30) and Rastawicki et al. (31). In the populations they examined, respectively: 531 vaccinated employees of a German hospital; 20,228 blood donors from Australia; 1,879 employees of a Warsaw hospital; and 140 health care workers (from different Warsaw hospital), also showed no statistically significant difference in immune response between the genders.

In contrast, different results were reached in works by: Romero-Ibarguengoitia et al. (32), Pellini et al. (33), and Swadźba et al. (34) in which respectively: 168; 248 and 100 adults from Monterrey (Mexico); Rome (Italy) and Krakow (Poland) were studied. In each of these articles, it was in adult women who responded to vaccination with the production of higher antibody titers.

Once again, further study on the topic of differences between genders in the production of anti-SARS-CoV-2 antibody is needed.

## 5 Limitations of the study

The study may be limited by the lack of information about vaccination or previous COVID-19 infection among study participants. We qualified every patient who was hospitalized for reasons other than COVID-19 infection. Therefore we did not take this information into account, which was one of the assumptions of the project.

We are aware that the size of the study groups in two voivodeships is different, which is also a result of the method of collecting the study population.

Some limitation may be the use of different analytical methods for determining anti-SARS-CoV-2 IgG antibodies. For this reason, in the analysis of the obtained results, we used the BAU/ml index, which is recognized by other researchers.

## 6 Conclusion

1. In the studied population, the concentration of anti-SARS-CoV-2 IgG antibodies differs significantly in people >60 years compared to the groups of people aged 18–35 and the group aged 36–60. However, the correlations between age and antibody concentration were significant but slight, so age should not be the primary determinant of the expected immune response after vaccination or COVID-19 disease.
2. Women and men presented a similar immune response to SARS-CoV-2 during the COVID-19 pandemic.
3. The number of infections among people in a specific time period influenced the number of patients to be tested for anti-SARS-CoV-2 antibodies.

The results of our study cover a small part of the entire population affected by the pandemic, but may be useful for preparing, e.g., meta-analysis. Control of the concentration of anti-SARS-CoV-2 antibodies allows for the assessment of acquired immunity in the population, which may be used in creating strategies for dealing with future pandemics.

## Data Availability

The raw data supporting the conclusions of this article will be made available by the authors, without undue reservation.
