# Supplementary figures and images for: Humoral response against COVID-19 in the population of western region of Poland
Source: Front Public Health. 2025 Aug 5;13:1648937. doi: 10.3389/fpubh.2025.1648937 (PMC12361232; doi:10.3389/fpubh.2025.1648937)

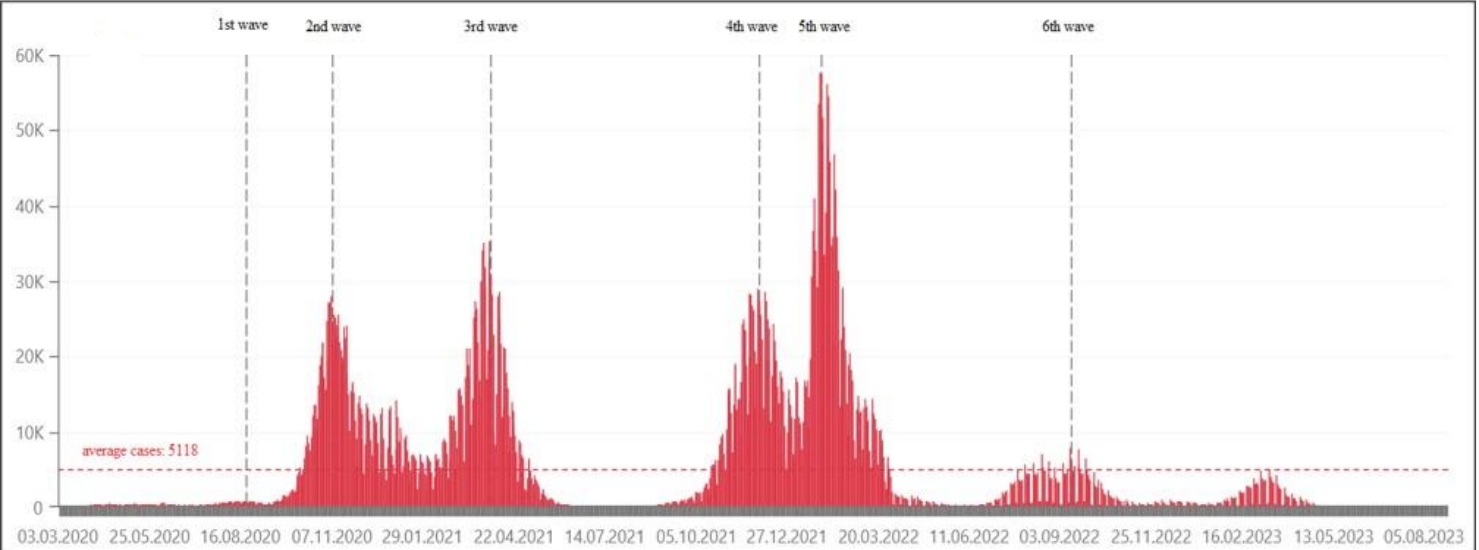

Supplement: Supplementary file 1 [file Data_Sheet_1.zip › Supplementary Material Presentation/Figure 1. A - Daily confirmed COVID-19 cases in Poland, from March 2020, in tens of thousands of people [4]..jpg]

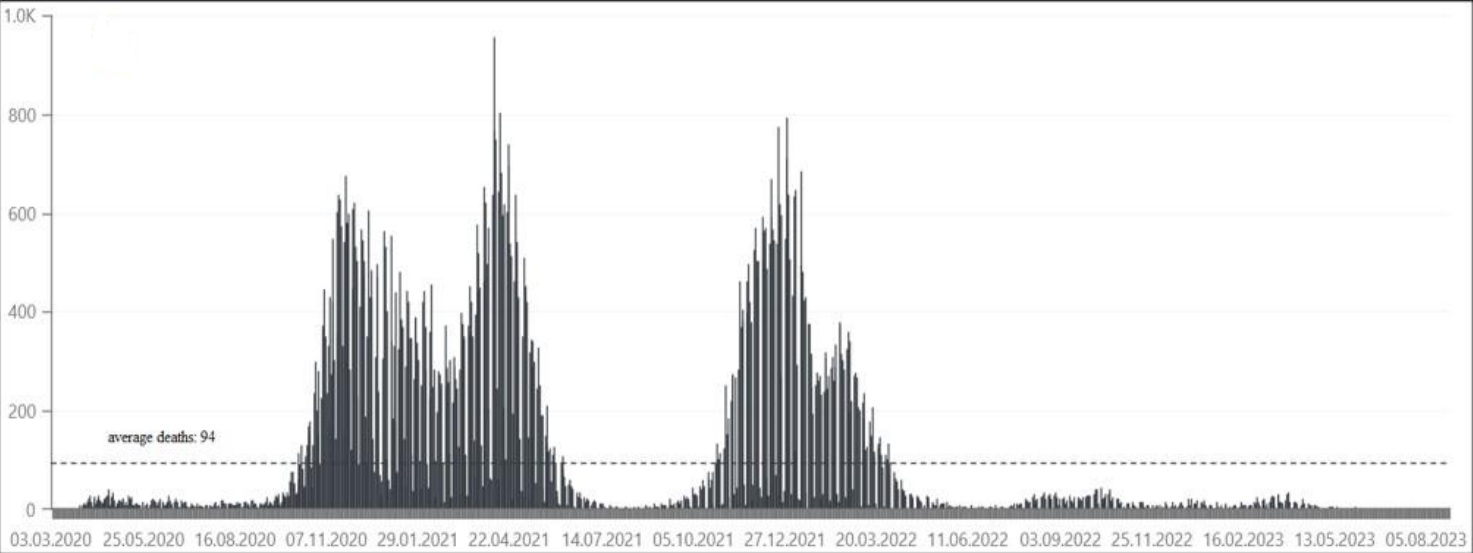

Supplement: Supplementary file 1 [file Data_Sheet_1.zip › Supplementary Material Presentation/Figure 1. B - Daily deaths due to COVID-19 in Poland, from March 2020, in hundreds of people [4]..jpg]

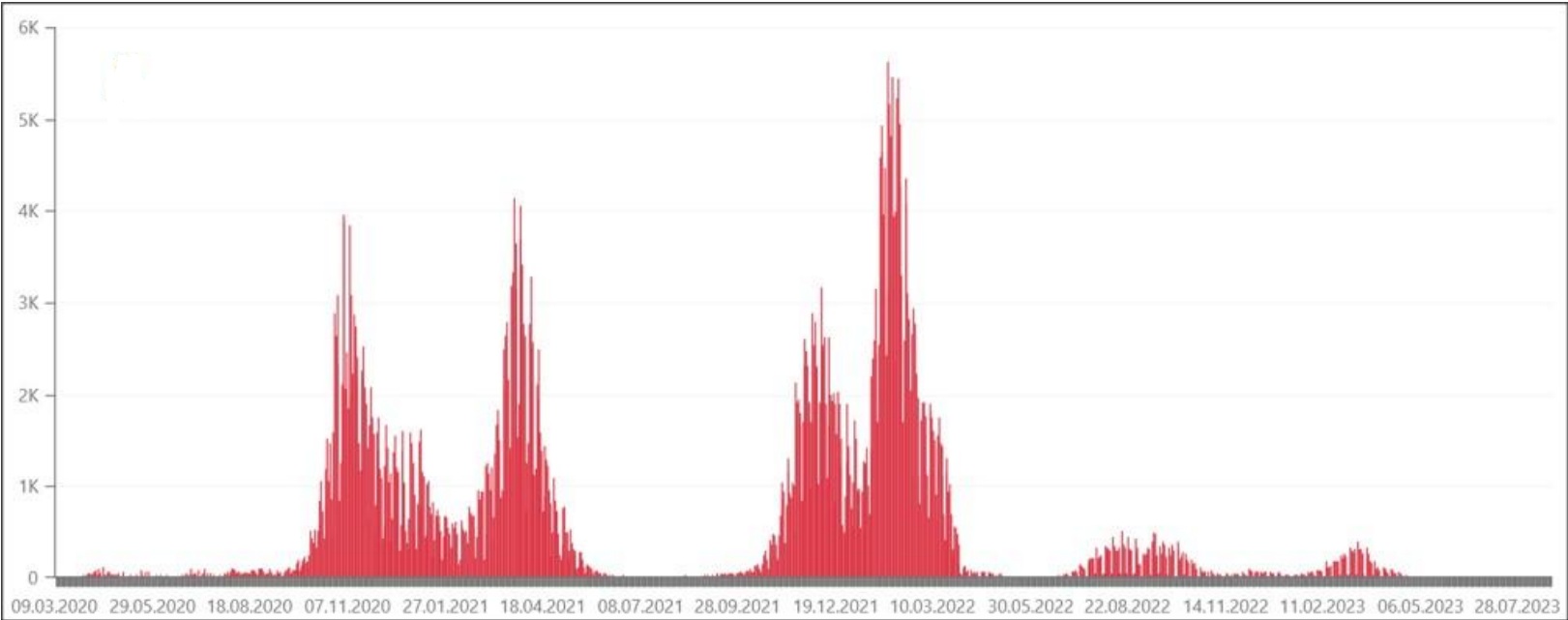

Supplement: Supplementary file 1 [file Data_Sheet_1.zip › Supplementary Material Presentation/Figure 2. A - Daily confirmed COVID-19 cases in the Wielkopolskie Voivodship, from March 2020, in thousands of people [5]..jpg]

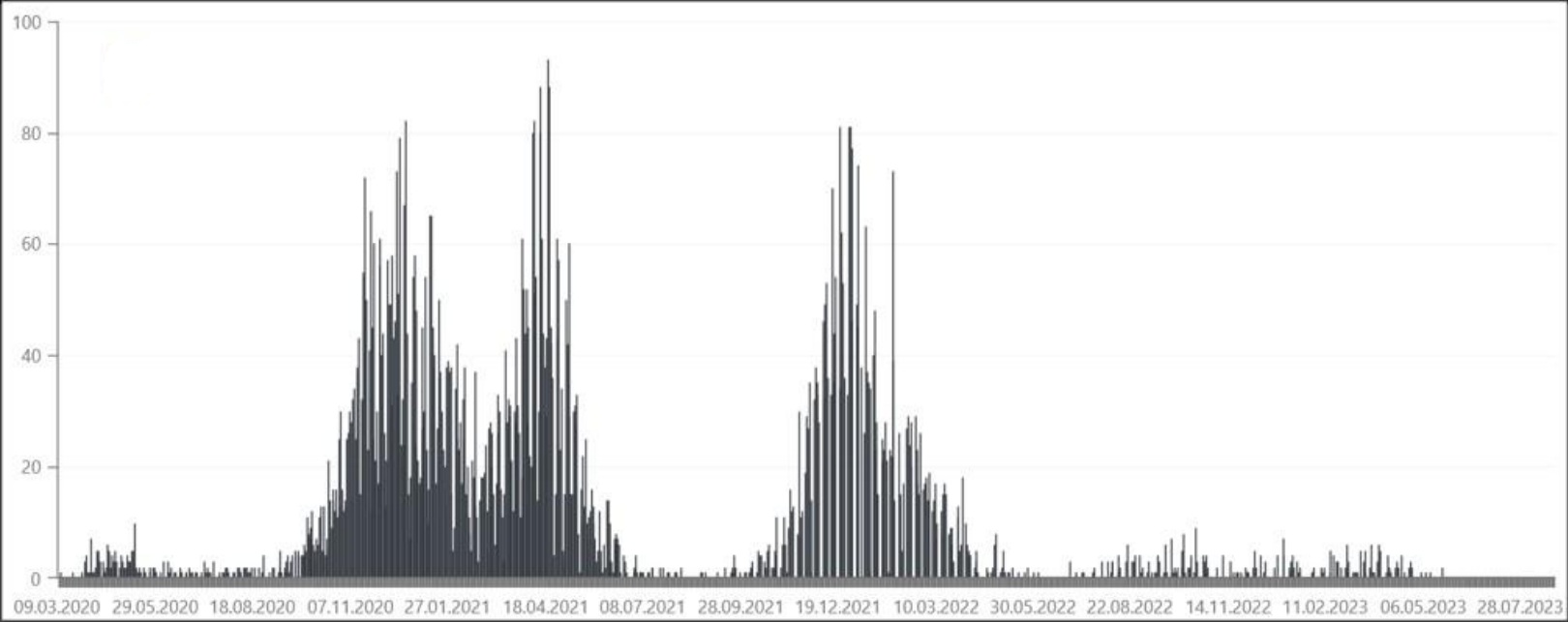

Supplement: Supplementary file 1 [file Data_Sheet_1.zip › Supplementary Material Presentation/Figure 2. B - Daily deaths due to COVID-19 in the Wielkopolskie Voivodship, from March 2020, in tens of people [5]..jpg]

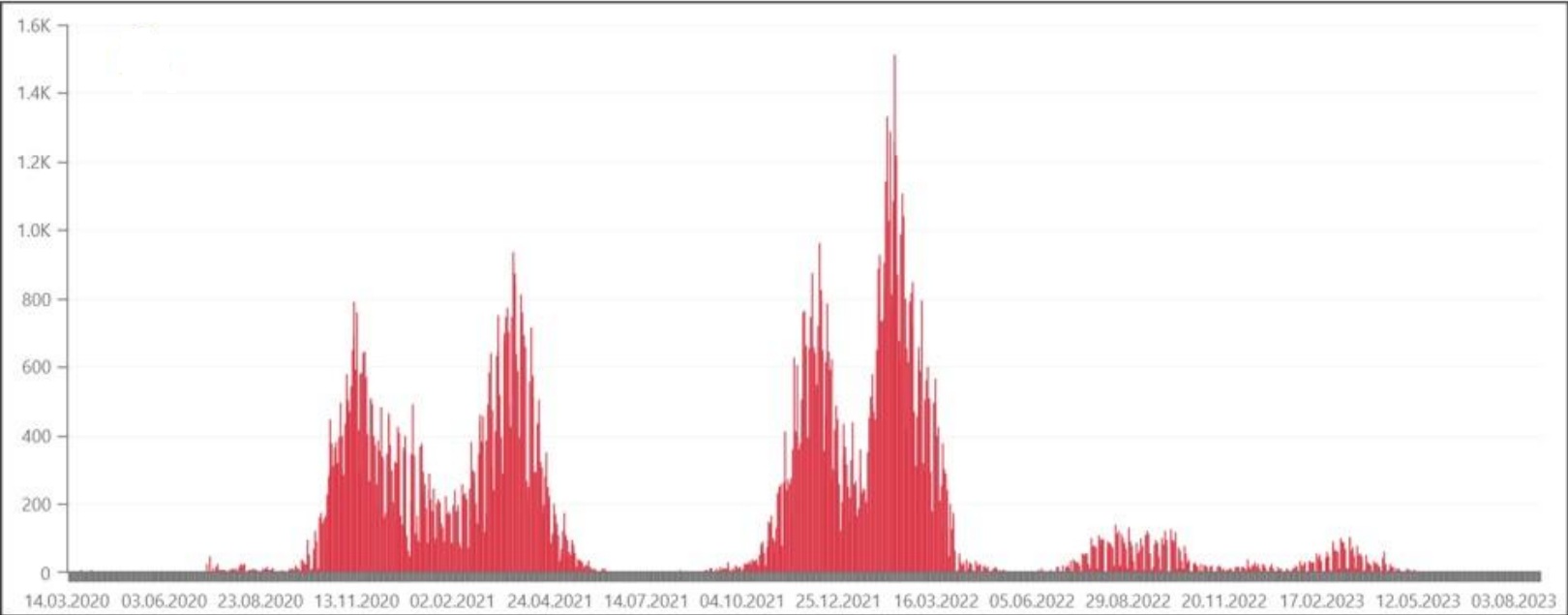

Supplement: Supplementary file 1 [file Data_Sheet_1.zip › Supplementary Material Presentation/Figure 3. A - Daily confirmed COVID-19 cases in the Lubuskie Voivodeship, from March 2020, in hundreds of people [6]..jpg]

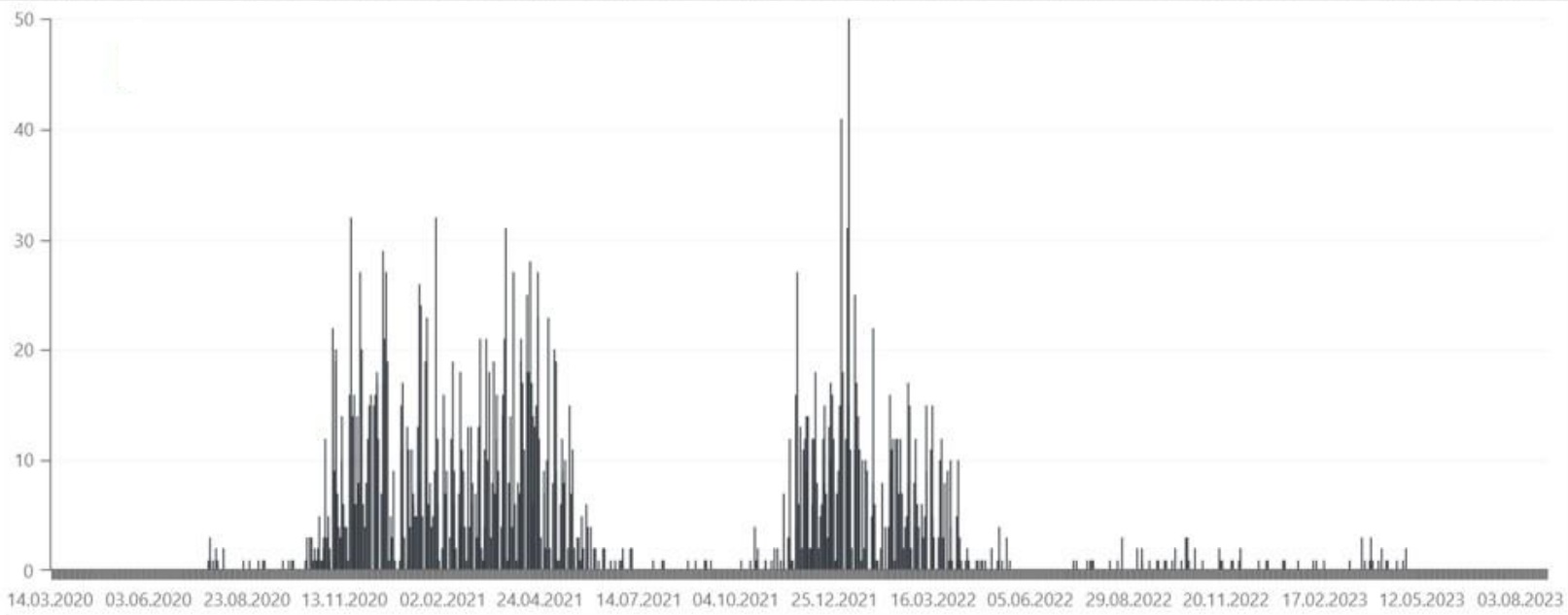

Supplement: Supplementary file 1 [file Data_Sheet_1.zip › Supplementary Material Presentation/Figure 3. B - Daily deaths due to COVID-19 in the Lubuskie Voivodeship, from March 2020, in tens of people [6]..jpg]

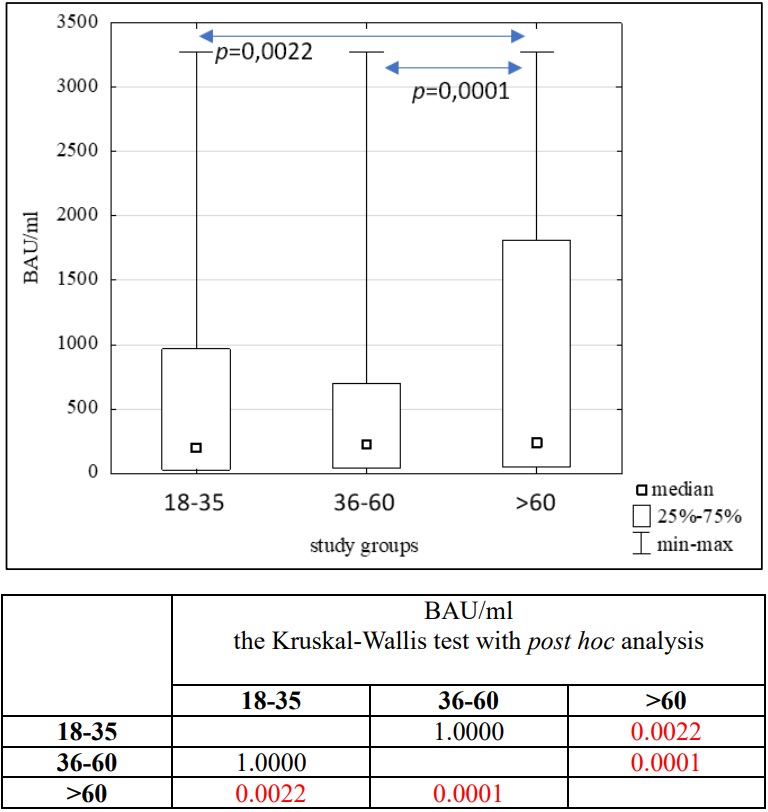

Supplement: Supplementary file 1 [file Data_Sheet_1.zip › Supplementary Material Presentation/Figure 4. The comparison of BAUml between age subgroups in the entire study population..jpg]

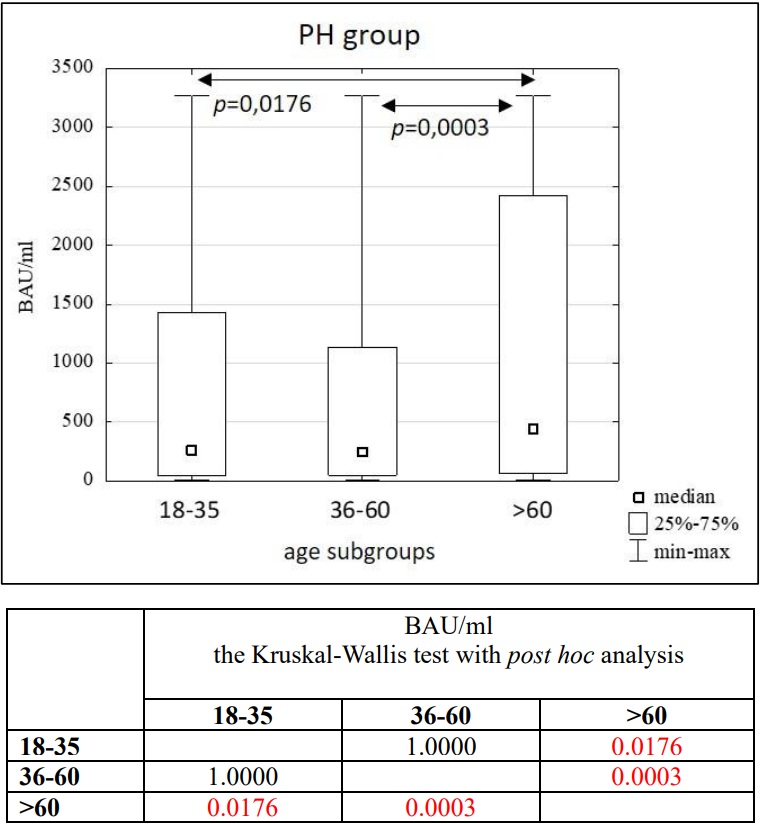

Supplement: Supplementary file 1 [file Data_Sheet_1.zip › Supplementary Material Presentation/Figure 5. The comparison of BAUml between age subgroups in the PH group..jpg]

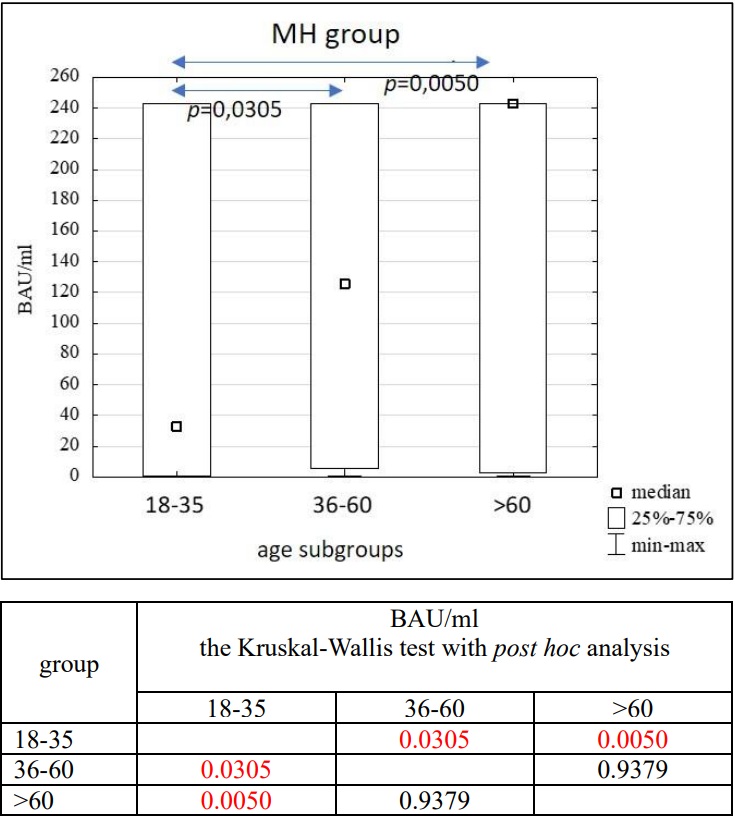

Supplement: Supplementary file 1 [file Data_Sheet_1.zip › Supplementary Material Presentation/Figure 6. The comparison of BAUml between age subgroups in the MH group..jpg]

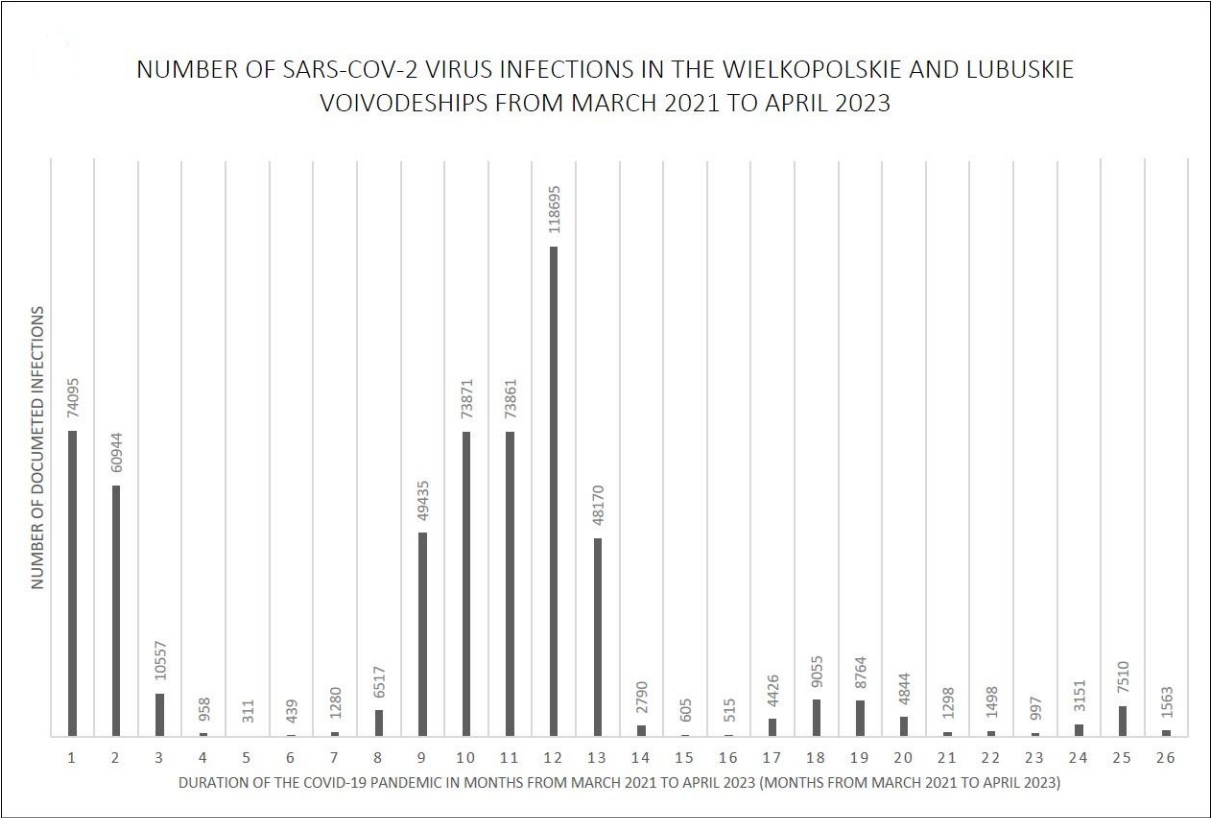

Supplement: Supplementary file 1 [file Data_Sheet_1.zip › Supplementary Material Presentation/Figure 7. A - The number SARS-CoV-2 virus infections in the Wielkopolskie and Lubuskie Voivodeships from March 2021 to April 2023..jpg]

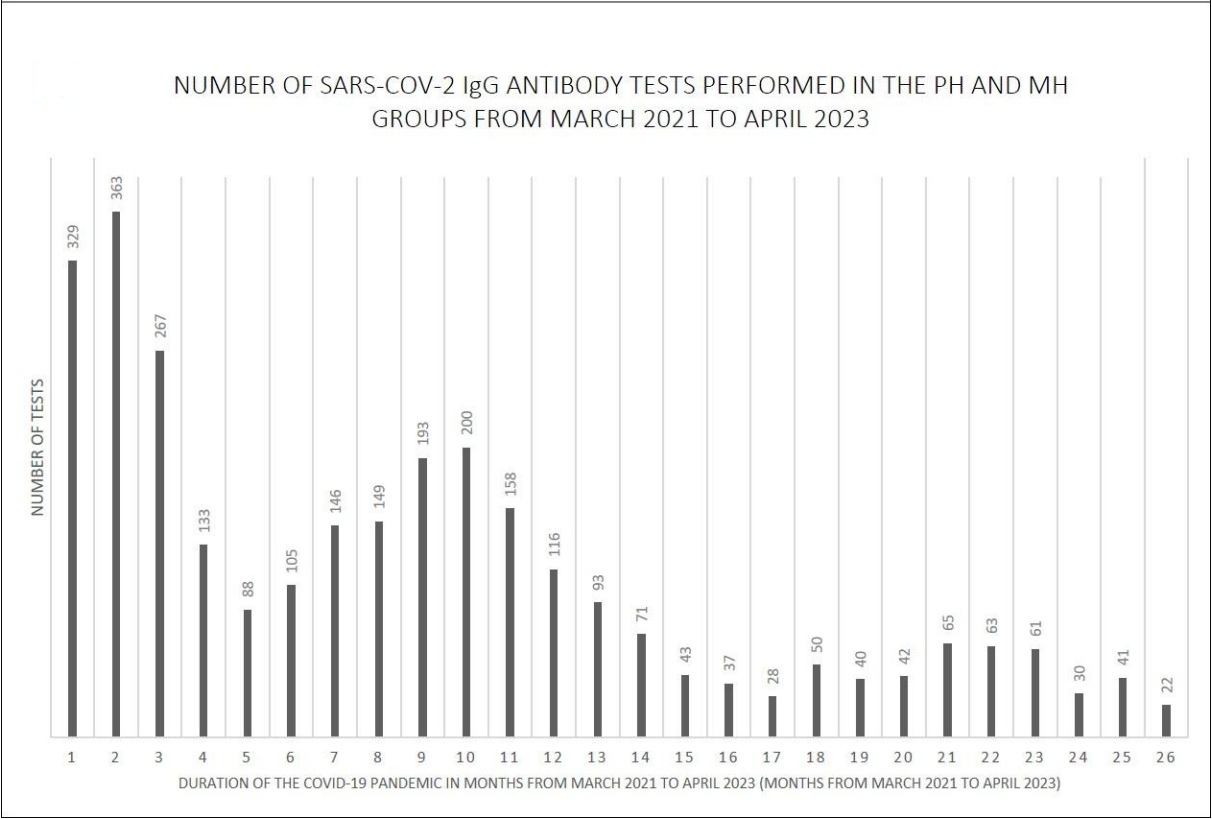

Supplement: Supplementary file 1 [file Data_Sheet_1.zip › Supplementary Material Presentation/Figure 7. B - The number SARS-CoV-2 IgG antibody tests performed in the PH and MH groups from March 2021 to April 2023..jpg]

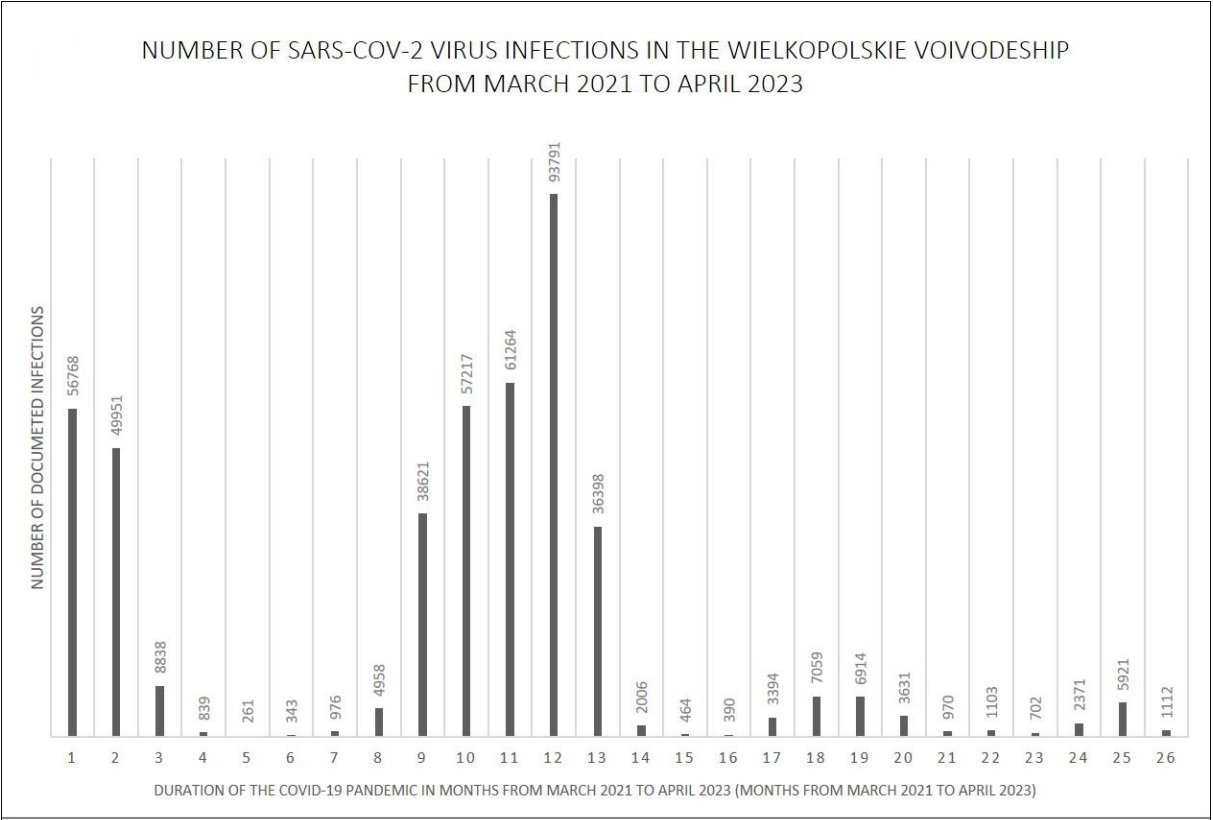

Supplement: Supplementary file 1 [file Data_Sheet_1.zip › Supplementary Material Presentation/Figure 8. A - The number SARS-CoV-2 virus infections in the Wielkopolskie Voivodeship from March 2021 to April 2023..jpg]

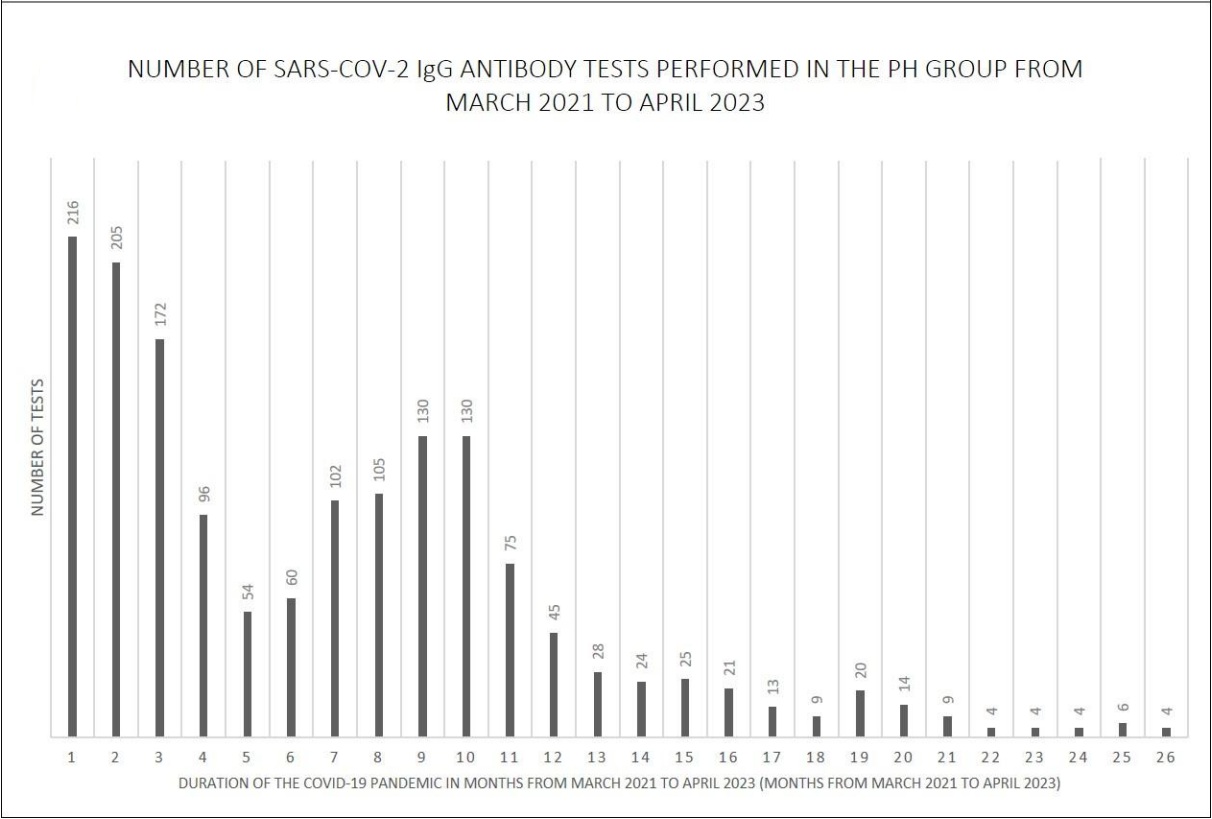

Supplement: Supplementary file 1 [file Data_Sheet_1.zip › Supplementary Material Presentation/Figure 8. B - The number SARS-CoV-2 IgG antibody tests performed in the PH group from March 2021 to April 2023..jpg]

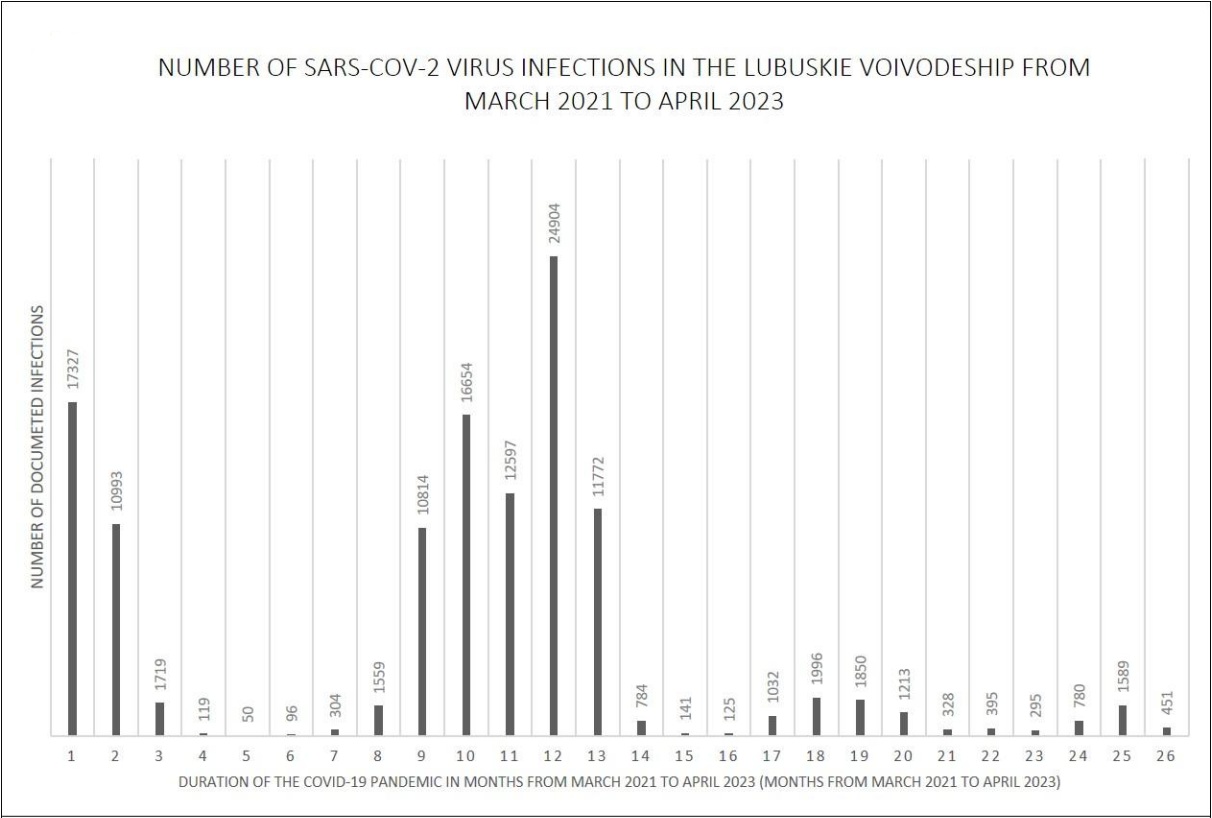

Supplement: Supplementary file 1 [file Data_Sheet_1.zip › Supplementary Material Presentation/Figure 9. A - The number SARS-CoV-2 virus infections in the Lubuskie Voivodeship from March 2021 to April 2023..jpg]

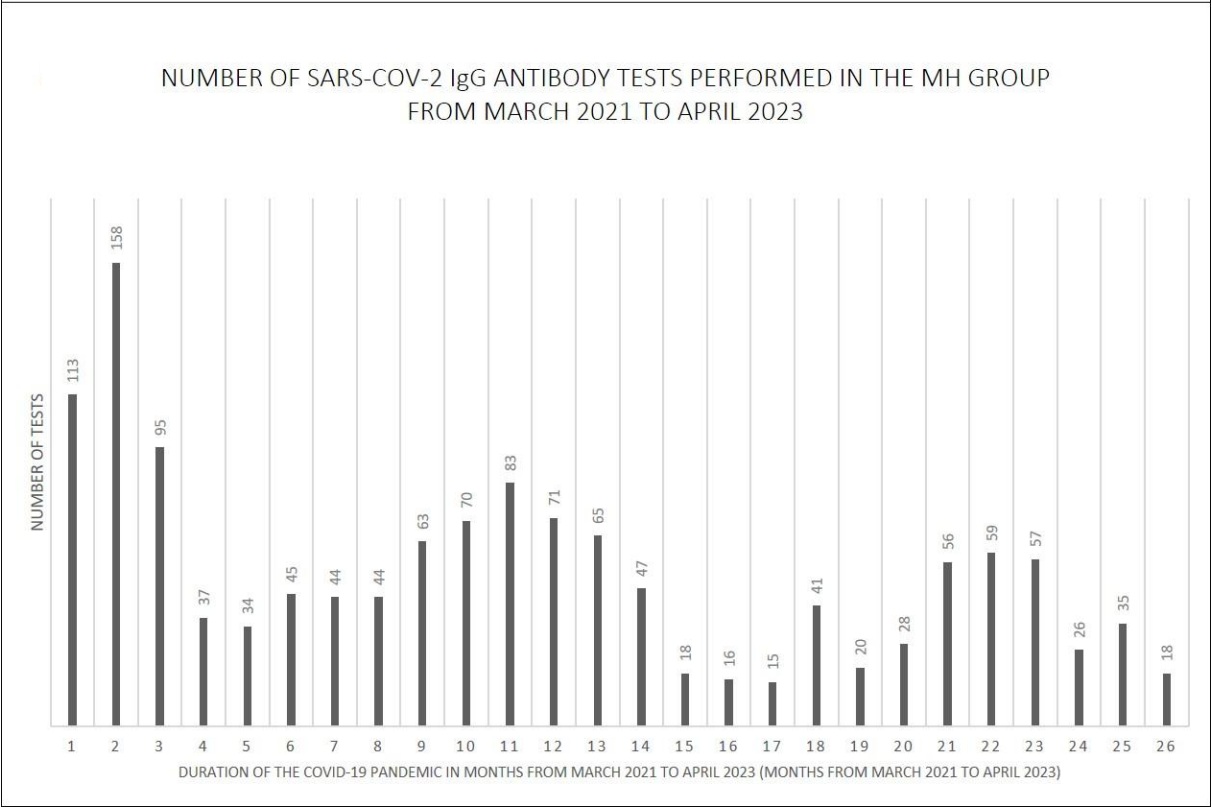

Supplement: Supplementary file 1 [file Data_Sheet_1.zip › Supplementary Material Presentation/Figure 9. B - The number SARS-CoV-2 IgG antibody tests performed in the MH group from March 2021 to April 2023..jpg]
